# Neglected etiologies of prolonged febrile illnesses in tropical and subtropical regions: A systematic review

**DOI:** 10.1371/journal.pntd.0011978

**Published:** 2024-06-21

**Authors:** Stefano Musumeci, Alexandra Kruse, François Chappuis, Tomas Ostergaard Jensen, Gabriel Alcoba

**Affiliations:** 1 Division of Infectious Diseases, Geneva University Hospitals (HUG), Geneva, Switzerland; 2 Médecins Sans Frontières (MSF) / Doctors Without Borders, Brussels, Belgium; 3 Department for Clinical Medicine, Copenhagen University, Copenhagen, Denmark; 4 Division of Tropical and Humanitarian medicine, Geneva University Hospitals (HUG), Geneva, Switzerland; 5 Centre of Excellence for Health, Immunity, and Infections, Rigshospitalet, Copenhagen University Hospital, Copenhagen, Denmark; 6 Médecins Sans Frontières (MSF) / Doctors Without Borders, Paris, France; 7 Médecins Sans Frontières (MSF) / Doctors Without Borders, Geneva, Switzerland; Institut Pasteur, FRANCE

## Abstract

**Background:**

Febrile illnesses that persist despite initial treatment are common clinical challenges in (sub)tropical low-resource settings. Our aim is to review infectious etiologies of “prolonged fevers” (persistent febrile illnesses, PFI) and to quantify relative contributions of selected neglected *target diseases* with limited diagnostic options, often overlooked, causing inadequate antibiotic prescriptions, or requiring prolonged and potentially toxic treatments.

**Methods:**

We performed a systematic review of articles addressing the infectious etiologies of PFI in adults and children in sub-/tropical low- and middle-income countries (LMICs) using the PRISMA guidelines. A list of *target diseases*, including neglected parasites and zoonotic bacteria (e.g., *Leishmania* and *Brucella)*, were identified by infectious diseases and tropical medicine specialists and prioritized in the search. Malaria and tuberculosis (TB) were not included as *target diseases* due to well-established epidemiology and diagnostic options. Four co-investigators independently extracted data from the identified articles while assessing for risk of bias.

**Results:**

196 articles from 52 countries were included, 117 from Africa (33 countries), 71 from Asia (16 countries), and 8 from Central and -South America (3 countries). *Target diseases* were reported as the cause of PFI in almost half of the articles, most frequently rickettsioses (including scrub typhus), relapsing fever borreliosis (RF-borreliosis), brucellosis, enteric fever, leptospirosis, Q fever and leishmaniasis. Among those, RF-borreliosis was by far the most frequently reported disease in Africa, particularly in Eastern Africa. Rickettsioses (including scrub typhus) were often described in both Africa and Asia. Leishmaniasis, toxoplasmosis and amoebiasis were the most frequent parasitic etiologies. Non-target diseases and non-tropical organisms (*Streptococcus pneumoniae*, *Escherichia coli*, and non-typhoidal *Salmonella spp)* were documented in a fifth of articles.

**Conclusions:**

Clinicians faced with PFI in sub-/tropical LMICs should consider a wide differential diagnosis including enteric fever and zoonotic bacterial diseases (e.g., rickettsiosis, RF-borreliosis and brucellosis), or parasite infections (e.g., leishmaniasis) depending on geography and syndromes. In the absence of adequate diagnostic capacity, a trial of antibiotics targeting relevant intra-cellular bacteria, such as doxycycline or azithromycin, may be considered.

## Author summary

“Prolonged” or “Persistent” Febrile illnesses (PFI) are common challenging diagnostic entities in LMICs. Symptoms and signs can be non-specific and diagnostic tests are often not available, and antibiotics inadequate. This systematic review aimed to investigate important causes of PFI in different geographical regions, as well as to highlight lack of data in these areas to stimulate discussion for future research. We found that zoonotic bacterial and neglected parasitic diseases accounted for substantial proportions of PFI cases, in particular rickettsiosis, borreliosis, brucellosis, leptospirosis, melioidosis, leishmaniasis, toxoplasmosis and amoebiasis. Although data is very limited for many regions, our results suggest that the geographical context can help inform clinicians on the likelihood of different infections in a patient with PFI.

## Introduction

Febrile illnesses remain important diagnostic challenges in tropical and subtropical areas in low-and-middle-income-countries (LMICs) [1].

Additionally the proportion of febrile illnesses that are of non-malarial origin has emerged clearly since the roll-out of malaria rapid diagnostic tests (RDTs) [2]. The clinical challenge becomes greater when fever persists with no response to widely administered empiric treatments.

The NIDIAG group (Syndromic approach to Neglected Infectious Diseases Diagnosis) [3] highlighted the fact that the *Persistent Fever Syndrome*, *or Persistent Febrile Illness* (PFI) called *Prolonged Fevers* by other experts [4], has rarely been studied in the tropics and recommended that clinical guidance tools should be developed. They hypothesized that these conditions are underestimated because they are often found in peripheral health centers with limited diagnostic options.

Defining persistent fever and its etiology in tropical and subtropical regions with limited resources is a difficult task for healthcare workers [5,6]. A “seven-day fever” definition of PFI was used by the NIDIAG group in Sudan, Cambodia, Nepal and the Democratic Republic of Congo, because most common mild viral illnesses usually recover spontaneously within one week, and the remaining are more likely to be due to bacterial or parasitic infections including NTDs such as visceral leishmaniasis and trypanosomiasis, and widely prevalent infections such as amoebiasis and enteric fever which are not included in the official WHO list of NTDs [5,7]. The same definition of Prolonged Fever of ≥7 days is used by experts in tropical and travel medicine from Oxford-Mahidol [8]. This 7-day cut-off is thus primarily based on generally accepted expert consensus.

Guidance for diagnostic work-up of fever of unknown origin often includes complex investigations such as imaging studies including CT, MRI, and PET-scans, bone-marrow examinations, multi-site cultures, and various serological and molecular tests [9]. These guidelines are therefore of limited use in the rural tropics where more sophisticated laboratory and radiological tools are not available or difficult to access [8,10,11].

The aim of this systematic review is to identify common—as well as rare but potentially severe—etiologies of PFI and to inform clinicians on clinical management decisions in settings with limited diagnostic options.

## Methods

We conducted a systematic review of the etiology of PFI, across tropical and subtropical regions using PubMed and following PRISMA guidelines (see S1 PRISMA Checklist). All study designs were evaluated, and the search did not include a specific duration of fever. Articles in English, French, Portuguese, or Spanish, published in 2000 or later were included, and no age filter was applied. Articles on COVID-19 and immunocompromised patients were excluded. The initial search was conducted in August 2021 and updated in November 2023.An additional specific aim was to quantify the relative contribution of selected *target diseases*. These were identified after consultation with experts in infectious diseases and tropical medicine using four criteria: 1) limited field diagnostic options, in terms of microbiological or radiological confirmation; 2) often prolonged and potentially toxic treatments; 3) expected sizeable contribution to the burden of PFI based on clinical experience in LMICs; and 4) expected paucity of publications justifying a prioritization of the conditions in the search.

Malaria and tuberculosis (TB), both well-described causes of PFI in many LMICs, were not included as *target diseases* due to well-established diagnostic options and effective epidemiological surveillance.

The final selection of *target diseases* used in the search, caused by either neglected and/or zoonotic pathogens, is listed in **Table 1**.

**Table 1:** *Target diseases* identified by expert consensus as important neglected causes of persistent febrile illness and prioritized in search terms.

Parasitic diseases	Amoebic abscess
	Babesiosis
	Chagas disease*
	Human African Trypanosomiasis (HAT)*
	Toxoplasmosis
	Visceral leishmaniasis (kala azar) *
Bacterial diseases	Bartonellosis
	Borreliosis (relapsing fever)
	Brucellosis
	Enteric fever
	Leptospirosis
	Melioidosis
	Q fever (coxiellosis)
	Rickettsiosis
	Scrub typhus
Viral diseases	Crimean-Congo hemorrhagic fever (CCHF)
	Lassa fever
	Rift Valley fever (RVF)
Fungal diseases	Systemic mycoses

*Included in the list of neglected tropical diseases by the WHO.

As shown in **Table 2**, three different clusters of search terms were developed. Cluster 1 (PFI and synonyms) was then combined with each of the other 2 clusters and applied as individual searches.

**Table 2:** Search terms used to identify causes of persistent febrile illness in tropical and subtropical regions, divided into three clusters.

Cluster	Search terms
1. Persistent febrile illness and synonyms	"Fever of Unknown Origin"[Mesh] OR "Relapsing Fever"[Mesh] OR « Persistent fever »[tw] OR « prolonged fever » [tw] OR « persisting fever » [tw] OR « constant fever » [tw] OR « relapsing fever » [tw] OR FUO OR « fever of unknown origin » [tw] OR « fever without source » [tw]
2. Geographical regions of interest	africa OR asia OR “south america “[tw] OR “latin America” [tw] OR “developing countr* “[tw] OR “middle east” [tw] OR “low income countr*”[tw] OR “low-middle income countr*”
3. Target diseases	parasit* OR “winterbottom’s sign” OR “mediterranean fever”[tw] OR borreliosis OR salmonellosis OR "pretibial fever"[tw] OR "Parasitic Diseases"[Mesh:NoExp] OR Leishmania[MeSH Terms] OR Leishmaniasis[Mesh] OR "Trypanosomiasis, African"[Mesh] OR Trypanosoma[Mesh] OR "Chagas Disease"[Mesh] OR Babesia[Mesh] OR Babesiosis[Mesh] OR Toxoplasma[Mesh] OR Toxoplasmosis[Mesh:NoExp] OR "Entamoeba histolytica"[Mesh] OR Amebiasis[Mesh] OR "Mycoses"[Mesh] OR Coccidioidomycosis[Mesh] OR "Cryptococcus"[Mesh] OR Histoplasma[Mesh] OR Blastomycosis[Mesh] OR Blastomyces[Mesh] OR "Arbovirus Infections"[Mesh] OR "Lassa Fever"[Mesh] OR "Brucella"[Mesh] OR Brucellosis[Mesh] OR Salmonella[Mesh] OR "Salmonella Infections"[Mesh] OR "Borrelia Infections"[Mesh] OR "Rickettsia Infections"[Mesh] OR "Orientia tsutsugamushi"[Mesh] OR "Melioidosis"[Mesh] OR "Leptospirosis"[Mesh] OR "Q Fever"[Mesh] OR "Bartonella Infections"[Mesh]

### Screening and extraction

**Fig 1:**
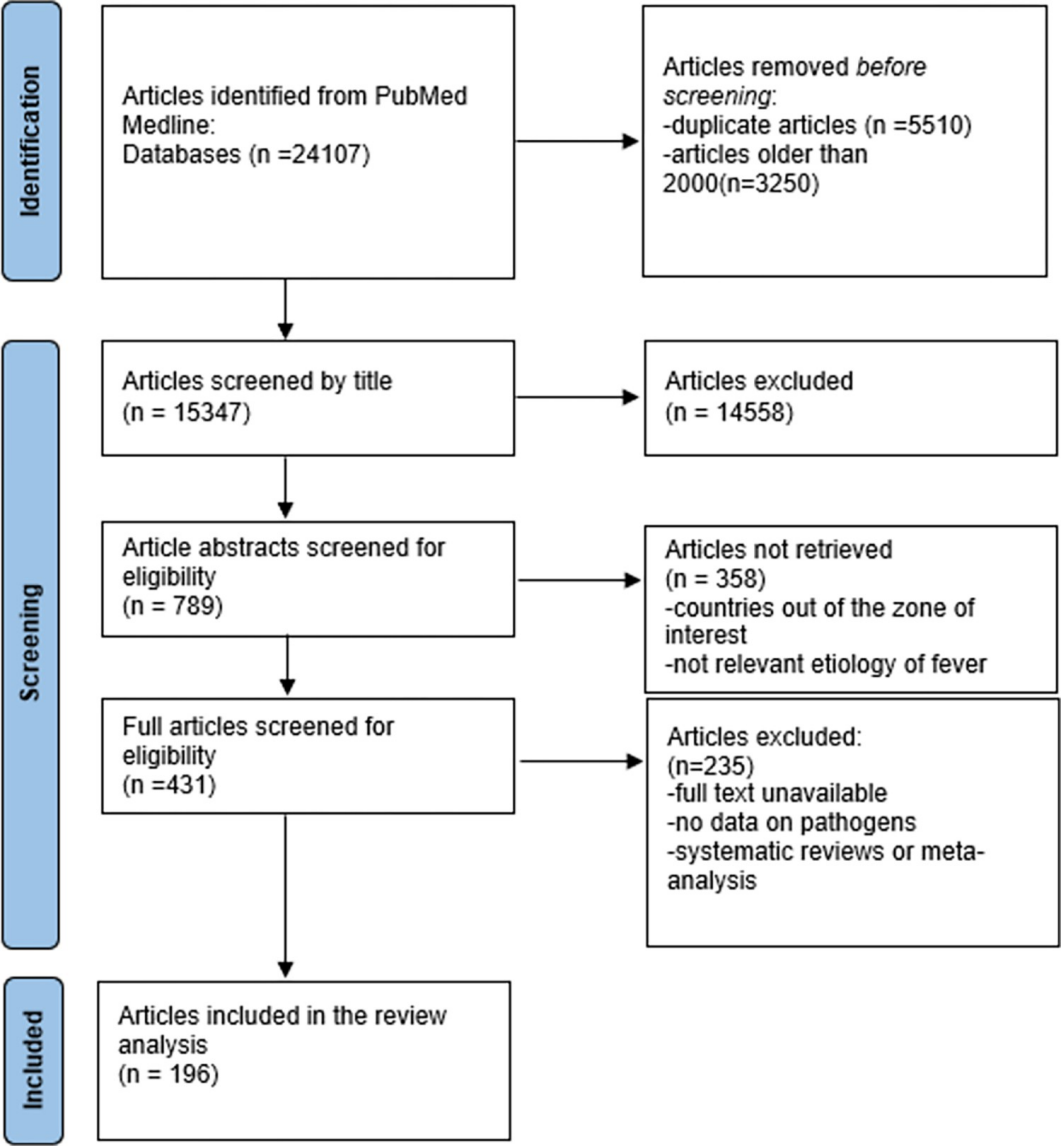
PRISMA flow diagram. Articles addressing either target diseases or other infectious causes of PFI in tropical and sub-tropical LMIC were included in the final list.

All articles initially identified were subsequently screened in 3 steps **(Fig 1).**

Articles not addressing our geographical area of interest and either *target diseases* or etiologies of PFI were excluded in all three steps.

In the first screening, by title, co-authors excluded articles addressing non-infectious causes of fever and research conducted in non-target regions. All study designs were evaluated, including narrative reviews, case-reports and case series on specific populations such as migrants and tourists as potential proxy sources to understand prevalence and etiology in certain areas, or those highlighting pathogens rarely described in a particular region.

The second screening by abstract, was done by two authors independently. In case of disagreement between both reviewers, we included the article if at least one of the reviewers considered it relevant. This was done to increase the sensitivity for *target diseases*.

In the third screening (full articles), each article was read in detail by one reviewer.

For all articles on the final list we performed an assessment of the quality of evidence by using the NIH study quality assessment tools [12]. We systematically extracted data on geographical region, type of study, study population, reported pathogens, and laboratory methods **(S1 Table)**.

Articles were categorized by continent and by region, according to the regions of the African Union for Africa [13], and the UN statistics agency [14] for Asia. As the number of articles from Asia was lower compared to the African continent, we decided to present them by proportionally similar macro-regions as follows: South- and Central Asia; East- and Southeast Asia; and West Asia. Central and South America were not divided into regions due to the paucity of articles found.

## Results

We identified 24,107 articles in the initial search, of which 435 were assessed for eligibility in the full-text step, and 196 were included in the final review **(S1 Table)**.

The 196 articles included in the review accounted for a population from 52 countries (33 from Africa, 16 from Asia, 3 from Central and South America). 93 articles (47%) included children from 0–5 years old, 105 articles (54%) included children and adolescents from 5–15 years old and 124 included adults (63%).

When laboratory techniques to identify pathogens were specified, polymerase chain reaction (PCR) was reported in 64 articles, microbiological culture in 32, serology (e.g., ELISA, immunoblot, RDT) in 101 articles, and direct microscopy in 25.

**Fig 2:**
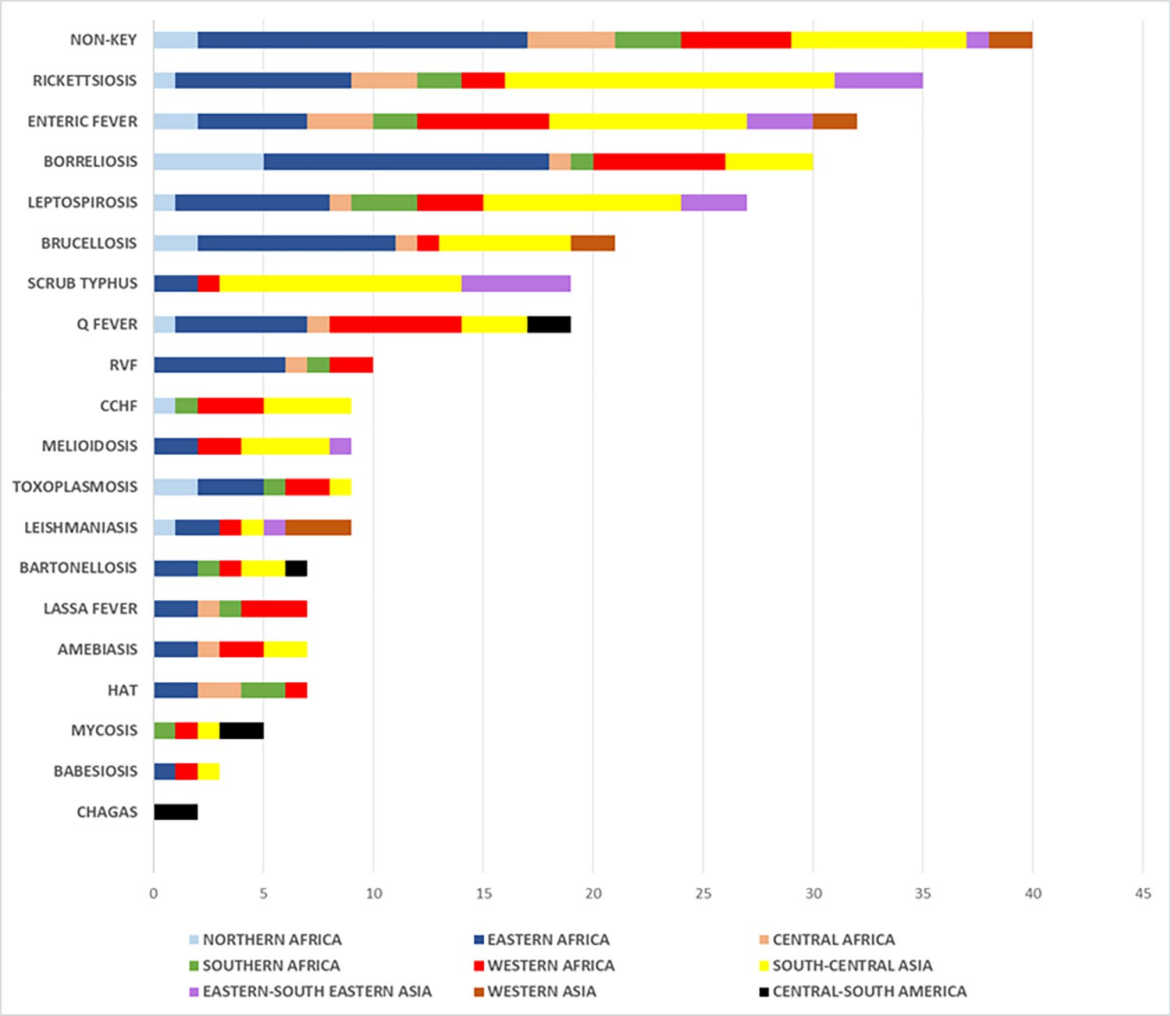
Number of articles identified in search and included in final review, stratified by pathogen and geographical region.

Overall, articles addressing conditions not on the list of *target diseases* accounted for a fifth (N = 40) of the total amount included in the review. Ubiquitous bacteria such as *Streptococcus pneumoniae*, *Escherichia coli*, *Salmonella* spp. (non-typhoidal) were commonly reported in association with PFI **(Fig 2)**.

Articles graded as high-quality evidence are discussed in more details in the following sections, and presented in **Table 3.** The initial search was conducted in August 2021 and to ensure inclusion of contemporary articles, we repeated the search and updated results in November 2023[5].

**Table 3:** Articles graded as high-quality evidence, containing prevalence data on etiologies of PFI.

Country/-ies	Sample (diagnostic tests)	Duration of fever (days)	Findings (etiology if available or clinical diagnosis)	Case (n/N)	(%)
**AFRICA**					
Kenya *(Maina et al*.*)* [20]	364 blood samples from febrile patients (serology)	*≥2*	*Rickettsia* spp. *Coxiella burnetii* *Orientsia* spp.	73/364 25/364 3/364	26% 8.9% 1.1%
Tanzania *(Crump et al*.*)* [18]	870 blood, urinary samples from febrile patients (culture, PCR, serology. antigen, blood smear, serology) * HIV prevalence <13 yo 12.2%; >13 yo 39%	*≥2*	**Bloodstream infections** **Bacterial** Mycobacteria*l Fungal * Plasmodium spp. **Zoonoses and arboviruses** *Coxiella burnetii* *Rickettsia* spp. (typhus group) Chikungunya virus *Rickettsia* spp. (spotted fever group) *Leptospira* spp. *Mycobacterium* spp.	85/870 14/870 25/870 14/870 40/453 36/450 55/700 24/482 16/435 2/450	**9.8%** 1.6% 2.9% 1.6% 8.8% 8% 7.9% 5% 3.5% 0.4%
Tanzania *(D’Acremont et al*.*)* [19]	1005 (culture, rapid test, serology, PCR and chest X-ray)	*Up to 7*	Acute respiratory infection Systemic infection (Viral causes 78%, common bacteriemia, *Rickettia* spp., etc..) Nasopharyngeal viral infection Malaria Gastroenteritis UTI, skin or mucosal infection Enteric fever		51% 11% 10% 9% 8% 6% 3%
CAR *(Gadia et al*.*)* [26]	198 serum samples from patients with fever, jaundice, and negative IgM for Yellow Fever (serology,PCR, rapid test)	*Up to 14*	HBV HEV HCV *Plasmodium falciparum* HDV	32/162 27/198 9/162 4/198 17/50	19.8% 13.6% 5.6% 2% 34%
Ethiopia (*Ibrahim et al*.*)*[39]	190 serum from healthy adults in close contact with livestock (serology)	*n/a*	*Coxiella burnetii* RVF *Brucella* spp.	50/188 25/190 5/178	26.3% 13.2% 2.8%
Egypt *(Kabapy et al*.*)* [16]	979 patients with FUO (extensive diagnostic work-up)	*31 ± 10*	**Infectious causes (79.1%, n = 774)** Respiratory tract infection Urinary tract infection *Brucella* spp. Enteric fever HCV Others	532/774 103/774 42/774 28/774 22/774 47/774	68.7% 13.3% 5.4% 3.6% 2.8% 6.2%
DRC *(Lunguya et al*.*)*[27]	3820 patients suspected of typhoid fever (serology, blood culture)	*n/a*	**Positive blood cultures (n = 363)** *Salmonella enterica serovar Typhi/Parathyphi* *Non-typhoid Salmonella spp*. *Klebsiella species* *Escherichia coli* *Enterobacter species* *Staphylococcus aureus*	92/363 134/363 57/363 35/363 25/363 20/363	25.3% 36.9% 15.7% 9.6% 6.9% 5.5%
Uganda *(Muloki et al*.*)*[21]	251 patients with prolonged fever (serology only for *Brucella spp*.)	*n/a*	Positive serology for *Brucella* spp.	47/251	18.7%
Namibia *(Noden et al*.*)*[29]	319 healthy blood donors (serology)	*n/a*	*Coxiella burnetii* *Rickettsia spp*. *(typhus group)* *Rickettsia spp*. *(spotted fever group)*	72/276 32/269 40/269	26.1% 11.9% 14.9%
Tanzania *(Reller et al*.*)*[17]	382 samples of 310 febrile patients (PCR, blood smear)	*n/a*	*Borrelia* spp. *Plasmodium spp*.	15/382 47/382	3.9% 12.3%
CAR (Rubbo et al.) [25]	497 serum samples from patients with fever, jaundice, and negative IgM for Yellow Fever (serology and PCR for *Leptospira spp*)	*n/a*	***Leptospira* spp. *diagnosed by*:** *ELISA and/or agglutination* *PCR*	46/445 0/497	10.3%
Morocco *(Sarih et al*.*)* [15]	127 patients with unexplained fever (PCR *Borrelia spp*)	*n/a*	*Borrelia* spp.	26/127	20.5%
Malawi *(Walsh et al)*[28]	365 positive blood cultures from neonates and children with FUO (blood culture)	*n/a*	*Non-typhi Salmonella* *Enteric Gram-negative bacilli* *Streptococcus pneumoniae* *Haemophilus influenzae* *Salmonella enterica serovar Typhi* *Streptococcus agalactiae* *Lancefield group D streptococci* *Staphylococcus aureus* *Streptococcus pyogenes* *Other*	140/365 91/365 59/365 21/365 15/365 13/365 10/365 7/365 6/365 3/365	38.4% 24.9% 16.2% 5.8% 4.1% 3.6% 2.7% 1.9% 1.6% 0.8%
Ethiopia *(Zerfu et al*.*)* [23]	630 people screened for febrile illness related symptoms (blood smear, serology)	*n/a*	*Rickettsia* spp. *Salmonella enterica serovar Typhi/Parathyphi* *Brucella* spp. *Plasmodium falciparum*	88/630 46/630 28/630 16/630	14% 7.3% 4.4% 2.5%
**ASIA**					
Nepal *(Thompson et al*.*)* [32]	627 blood samples form febrile patients (blood culture,PCR, serology)	*>3*	*Salmonella enterica* serovar Typhi/Parathyphi *Rickettsia* spp. *Hantavirus* *Coxiella burnetii*	218/617 24/125 2/125 1/125	34.8% 19.2% 1.6% 0.8%
Laos *(Chansamouth et al*.*)*[37]	250 blood samples from febrile pregnant women (blood culture, serology, antigen, PCR)	n/a	Dengue virus *Rickettsia* spp. *Orientsia* spp. *Salmonella enterica serovar Typhi* *Mycobacterium tuberculosis* *Staphylococcus aureus* *Leptospira* spp. Japanese Encephalitis virus *Plasmodium falciparum*	132/250 10/250 9/250 6/250 2/250 1/250 1/250 1/250 1/250	53% 4% 3.6% 2% 0.8% 0.4% 0.4% 0.4% 0.4%
Myanmar *(Elders et al*.*)* [38]	700 blood samples (serology)	*n/a*	*Orientsia* spp. *Rickettsia* spp. *(spotted fever group)* *Rickettsia* spp. *(typhus group)*	133/700 35/700 21/700	19% 5% 3%
India *(Mittal et al*.*)* [33]	824 patients with FUO for which enteric fever and malaria were ruled out (serology)	*n/a*	*Rickettsia* spp.	67/824	8.2%
Sri Lanka *(Premaratna et al*.*)* [35]	28 patients with FUO improved on doxycycline (serology)	*>7*	*Rickettsia* spp. *(spotted fever group)* *Orientsia* spp.	11/28 10/28	39% 35%
Bangladesh *(Rahman et al*.*)* [31]	300 samples from febrile patients (serology, PCR for *Brucella* spp)	*>21*	**Positive serology for *Brucella* spp.** Positive PCR.*among positive serology*	6/300 0/6	2%
India *(Rizvi et al*.*)* [30]	357 febrile patients for which malaria, enteric fever, and leptospirosis were ruled out (seroloy *Orientsia* spp.)	*>5*	*Orientsia* spp.	91/357	25.5%
India *(Sankar et al*.*)* [34]	305 blood, sputum samples from febrile patients (culture, PCR, serology)	*≥5*	*Mycobacterium tuberculosis* *Burkholderia pseudomallei Salmonella enterica* serovar Typhi/Parathyphi	21/350 14/350 11/350	6.8% 4.5% 3.6%
Nepal *(Tamrakar et al*.*)*[36]	5736 patients with suspected enteric fever from 2 communities (blood culture)	*n/a*	*Salmonella enterica serovar Typhi/Parathyphi*	304/5736	5.3%
**MULTI-COUNTRY**					
Sudan, DRC, Nepal, Cambodia *(Bottieau et al*.*)* [5]	1922 blood and urine samples from febrile patients (>5 yo) (serology, culture, blood smear, PCR, antigen)	*≥7*	*Plasmodium* spp. *Mycobacterium tuberculosis* *Leishmania* spp. *Leptospira* spp. *Rickettsia* spp. *Salmonella enterica serovar Typhi/Parathyphi* *Brucella* spp. *Burkholderia pseudomallei* *HIV/opportunistic infections (other than TB)* *Borrelia* spp. *Entamoeba histolytica* *Trypanosoma brucei gambiense*	154/1922 129/1922 119/1922 77/1922 44/1922 34/1922 28/1922 16/1922 14/1922 12/1922 12/1922 2/1922	8% 6.7% 6.2% 4% 2.3% 1.8% 1.5% 0.8% 0.7% 0.6% 0.6% 0.1%
Senegal, Mauritania, Mali *(Vial et al*.*)*[24]	1 286 045 person-days of surveillance (presenting with fever) (blood smear, PCR)	*n/a*	*Average incidence of RF borreliosis infections*	11 per 100 person-years	n/a

### AFRICA

#### Northern Africa

13 articles from this region were included. RF-Borreliosis was the most described disease (N = 5), ahead of enteric fever, toxoplasmosis, brucellosis, and pathogens not included as *target diseases* (N = 2 each).

In Morocco a retrospective study including 127 patients with non-malarial PFI described *Borrelia hispanica* in 20% of patients detected by molecular testing (16S rRNA) in blood as a single etiology of PFI [15]. In a hospital-based study in Egypt on 979 patients (children and adults) with PFI, an infectious cause was found in 63.4% of cases. However, no cases of RF-borreliosis were found and brucellosis and enteric fever were the top PFI etiologies accounting for 4.3% and 2.9% respectively [16].

#### Eastern Africa

Eastern Africa (Tanzania, Kenya, Uganda, Ethiopia) accounted for most articles in Africa (n = 53). Tanzania was the country included in the highest number of articles. RF-borreliosis was the most frequently reported disease (n = 13), followed by brucellosis (n = 9), rickettsiosis (n = 8), Q fever and Rift Valley fever (n = 6 each). Reller et al. [17] designed a multiplex quantitative PCR (qPCR) assay to distinguish relapsing fever caused by *Borrelia* spp. from *Plasmodium falciparum* and *Plasmodium vivax* and applied it in a large cohort in Tanzania. Of 310 febrile patients, 13 (4.2%) had positive *Borrelia* spp. DNA.

Crump et al. prospectively studied 870 patients (both children and adults) with fever in Northern Tanzania, finding that malaria was clinically diagnosed in 528 patients (61%), but microbiologically was the actual cause of fever only in 14 cases whereas bacterial bloodstream infections (including *Salmonella* spp.) accounted for 85 cases confirmed by blood culture [18]

Among 1005 febrile children with fever lasting up to one week included in a study by D’Acremont et al. at two different sites in Tanzania, respiratory infections (either bacterial or viral) accounted for 51% of the etiologies. Interestingly, among 11% of the cohort that presented with systemic infections other than malaria and enteric fever, 78% were caused by human herpesvirus 6 (HHV-6) while the remainder were related to other viruses as well as bacteria including *Rickettsia* spp., *Leptospira* spp., and *Coxiella* spp [19].

Maina et al. investigated 370 febrile children in western Kenya and found that a sizeable proportion were seropositive for spotted fever group *Rickettsia* (22.4%), *Coxiella burnetii* (8.9%), *Orientia Tsutsugamushi* (3.6%, usually in Asia), and typhus group *Rickettsia* (1.1%) [20].

In Northern Uganda, seroprevalence of brucellosis, either with i-ELISA or STAT titres (>1:320), was 18.7% in a sample of 251 patients (children older than 5 years old and adults) with PFI [21].

In a seroprevalence study in Somali region (Ethiopia), aiming to establish seroprevalence of zoonotic diseases (including brucellosis, Q-fever and Rift Valley fever (RVF)) in humans and livestock by Rose Bengal and indirect ELISA, Ibrahim et al. found a positive results in 26.3% (n = 50) of human participants for Q fever, 13.2% (n = 25) for RVF, and 2.8% (n = 5) for brucellosis [22].

In the Afar Region (Ethiopia) among 630 people with febrile illness, *P*. *falciparum* was detected in only 2.5% of cases whereas seropositivity for *S*. *Typhi*, *Rickettsia* spp. and *Brucella* spp. was found in 7.3%, 14% and 4.4% [23].

#### Western Africa

27 articles were included mainly addressing enteric fever (n = 7), *Borrelia* spp. (n = 6) and non-*target diseases* (n = 6).

In rural areas of Senegal, Mauritania and Mali, Vial et al. found a high incidence of RF-Borreliosis of 11 per 100 person-years (diagnosed by PCR), suggesting that RF-Borreliosis is a common cause of fever in this region [24].

#### Central Africa

12 articles were included from this region (Central African Republic [CAR] and the Democratic Republic of Congo [DRC]), reporting on non-*target diseases* (n = 4), as well as the *target diseases* enteric fever (n = 3), rickettsiosis (n = 3), and HAT (n = 2).

In a retrospective study [25] from CAR, including 497 patients with fever and jaundice but negative for yellow fever virus, 46 had a positive ELISA test for leptospirosis, confirming that this disease is present in the country although in a retrospective serological assessment from 2008–2010 on 198 patients with febrile jaundice by Gadia et al. [26], none were positive for *Leptospira* spp.

Regarding enteric fever, Lunguya et al. highlighted that clinicians in DRC rely highly on the Widal test for diagnostic confirmation, although this test was often poorly performed and interpreted. Two case reports addressed Human African Trypanosomiasis in the region [27].

#### Southern Africa

Ten articles from Southern Africa (South Africa and Malawi) were included. Non-*target diseases* (n = 3) and leptospirosis (n = 3) were most frequently reported.

In a reference hospital in Malawi [28], 2123 blood cultures from febrile children (0–5 years old) were performed. Out of 365 positive (17%) blood cultures, non-typhoidal *Salmonella*, other enteric gram-negative bacilli and *Streptococcus pneumoniae* were the most common pathogens (38.4%, 24.9%, and 16.2% of isolates respectively).

In a study of 319 blood donors performed in Namibia to better understand the impact of zoonotic bacterial diseases as a cause of febrile illness, *C*. *burnetii* prevalence was 26.1%, and prevalence rates of 11.9% and 14.9% for spotted fever group and typhus group *Rickettsiae*, respectively [29].

### ASIA

69 articles from Asia, mainly India (n = 19), Bangladesh (n = 10) and Nepal (n = 9), were included.

#### South- and Central Asia

52 articles were included. Rickettsiosis (n = 17), scrub typhus (n = 11), leptospirosis (n = 9), enteric fever (n = 9) and non-*target diseases* (n = 8) were most frequently described.

Among 357 patients in India with *fever of more than 5 days*, 25.5% were positive for scrub typhus by IgM ELISA, after ruling out malaria, enteric fever and leptospirosis [30].

Rahman et al. [31] found 2% seroprevalence of brucellosis among patients in Bangladesh with *prolonged fever* (duration of fever not specified). Enteric fever was microbiologically confirmed by blood culture in 34.8% of 627 patients (age-groups not available) presenting with fever to a tertiary referral hospital in Nepal [32].

In Delhi (India), among serum collected from 824 patients with fever of unknown origin after ruling out malaria and enteric fever, 8.2% of seropositivity for *Rickettsia* spp. was was detected [33].

Sankar et al. [34] developed and evaluated a multiplex nested PCR for the simultaneous detection of three pathogens in 305 patients (children and adults) with *fever of unknown origin* in India. PCR from buffy coat samples was positive for *S*. Typhi in 10 individuals (3.3%), *B*. *pseudomallei* in 10 individuals (3.3%), and *M*. *tuberculosis* in 18 individuals (5.9%). Among 28 febrile patients in Sri Lanka with *fever persisting for more than 7 days*, who responded to empirical treatment of doxycycline, 11 (39%) patients were confirmed by serology (IgM, IgG) as having spotted fever group rickettsioses and 10 (36%) as having *Orientia tsutsugamushi* infection [35].

Tamrakar et al. studied 5,736 patients with suspected enteric fever from 2 diverse communities in Nepal (Kathmandu and Kavrepalanchok) and detected *S*. Typhi in 5.3% by microbiological culture. Adjusted enteric fever incidence in Kathmandu was 484 per 100 000 person-years and 615 per 100 000 person-years in Kavrepalanchok [36].

#### East and Southeast Asia

From this region 11 articles (Laos, Myanmar, Cambodia) were included. Scrub typhus (n = 5), rickettsiosis (n = 4), enteric fever (n = 3) and leptospirosis (n = 3) were the most reported.

In Laos, a study published by Chansamouth et al. [37] on 250 pregnant women presenting with PFI (4–7 days of illness after hospital admission), RDT (IgM), micro-immunofluorescence assays (IFA) and PCR for scrub typhus and murine typhus were performed, in addition to culture of blood and urine and clinical evaluation. Dengue (53%) and pyelonephritis of unknown microbiological etiology (30%) were the most diagnosed diseases. Murine typhus (4%), scrub typhus (3.6%) and enteric fever (2%) were also found. 17 women (7%) had more than one diagnosis.

In a study from Myanmar [38], 700 blood samples from patients of all ages in seven regions of the country were screened for IgG antibodies for scrub typhus, typhus group and spotted fever group *Rickettsia* by ELISA. Overall IgG seroprevalence for scrub typhus was 19%; 5% and 3% respectively for the two latter *Rickettsia* groups.

#### Western Asia

Five articles from Western Asia (Iraq, Syria) were included. Visceral leishmaniasis was the most frequently described condition (n = 3), ahead of enteric fever, brucellosis, and non-*target diseases* (n = 2 each). No articles were graded as high quality.

#### Central and South America

Only 4 articles were identified (Brazil, Mexico, Peru), reporting on systemic mycoses and Chagas disease (n = 2 each). Due to the scarcity of data and low quality of evidence, further analysis could not be performed for these regions.

## Discussion

This systematic review is to our knowledge the first to focus on the importance of neglected, but potentially severe, infectious etiologies of PFI in tropical and sub-tropical areas of the world with limited access to diagnostic tools. Globally, rickettsiosis, enteric (typhoid) fever, borreliosis, and brucellosis were the most reported bacterial diseases. RF-borreliosis was one of the most described *target diseases* in Africa with most articles from Eastern Africa.

Nevertheless, only few articles contained data comparing relative prevalence of infections within the same study, so cautious interpretation of these results is warranted. In the NIDIAG study, a comparative multi-organism diagnostic study, malaria (8%), TB (6.7%), leishmaniasis (6%), leptospirosis (4%), enteric fever (1.8%) and brucellosis (1.5%), were the top 6 etiologies of PFI, whereas RF-borreliosis only accounted for 0.6% of total identified causes of PFI.

Regarding other *target diseases*, rickettsioses (spotted group *Rickettsia africae*, *R*. *conori* and many others) were frequently described in both Asia and Africa. Interestingly scrub typhus (*Orientia tsutsugamushi)*, formerly thought to be geographically restricted to Asia, has also been demonstrated in Africa [18], South America, and a case has been described in the Middle-East with a new species, *Orientia chuto* [40]. Similarly, the epidemiological distribution of melioidosis (*Burkholderia pseudomallei*) has long been considered restricted to Australia and South-East Asia, but the disease is now reported across Asia and on the African continent, highlighting the need to consider ceftazidime or carbapenems in patients with PFI, risk factors for melioidosis (e.g., diabetes, post-floods, immunosuppression) and/or severe clinical presentation (e.g., sepsis) [41].

Enteric fever as well as zoonotic diseases such as leptospirosis, brucellosis, and Q fever, were demonstrated in most regions. These diseases require diagnostic tools that are often not available in rural areas of LMICs, although available in urban hospitals. The emergence of resistant strains (e.g., enteric fever) may limit therapeutic options further. This evidence should trigger more research and development into RDTs, beside serology, PCR, and simplified cultures and bacteriology kits.

Non-*target diseases* accounted for an important number of references. Common causes of acute febrile illnesses can also persist for seven days or more, including malaria, arboviruses (e.g. dengue), respiratory viruses, and globally distributed bacterial infections (*Staphylococcus aureus*, *Streptococcus pneumoniae*, *Escherichia coli*, non-typhoidal *Salmonella* spp, etc.). The latter can also cause suppurative complications such as endocarditis, empyema, abscess, all causing prolonged fevers, but we lack epidemiological data on these conditions. Limited access to imaging studies (ultrasound and echocardiography, plain X-rays, CT scan, MRI) and laboratory capacities likely contributes to an important gap in evidence and may cause an underestimation of the frequency of certain diseases in specific areas. On the other hand, restricted access to high quality diagnostic testing and a resulting dependency on serologic testing with poor specificity may also overestimate the burden of certain diseases.

When an unusual cause of PFI is suspected, selecting the appropriate therapy is certainly challenging and often incorrect. Clinicians and protocols tend to oversimplify and underestimate the variety of differential diagnoses. Whether replacing a failing empiric regimen or adding to already prescribed antibiotics will be an individualized decision that depends on the severity of illness. Being able to cease an ongoing failing regimen decreases risk of toxicity and potential risk of resistance development. Improved access to diagnostic tests will facilitate this decision.

Our review has important limitations: 1) The choice of *target diseases* was based on a consensus among co-investigators (focused on neglected diseases), as well as on the NIDIAG multi-country diseases selection [3]. This together with our overall screening process may have introduced some selection bias and excluded other potentially relevant pathogens; 2) Access to diagnostic tests, such as blood culture to diagnose enteric fever and brucellosis, was not possible in many of the studies and this could have introduced bias towards underestimating these 2 conditions; 3) Available published evidence may also be biased towards pathogens that are of particular interest to regional or global researchers and institutions (investigator bias). This could for example explain the large amount of data on RF-borreliosis compared to enteric fever in African regions. 4) Acknowledging that the 7-day PFI definition is not yet a globally accepted clinical entity, and to increase sensitivity for articles on *target diseases*, we did not include a specific duration of fever in our search, and this may have introduced heterogeneity when comparing studies [3,5,21,31]. Finally, although outside of the scope of our analysis, non-infectious causes of prolonged fever are likely to be of increasing relevance in LMICs, potentially with many of the same challenges with limited access to diagnostic options.

Internal validity was assured by an agreed methodology, selection by four investigators (at abstract and article level), to minimize subjectivity and maximize consistency of interpretation. In contrast, the relative paucity of data and predominance of publications from Africa, makes external validity and generalizability partly problematic. Variations in epidemiology can happen over short distances and data from one country or region cannot necessarily be extrapolated to neighboring countries or regions.

## Conclusions

Clinicians confronted with PFI in tropical or subtropical LMICs, should focus on treating common bacterial infections while keeping other key etiologies in their differential diagnosis of PFI: enteric (typhoid) fever, zoonotic and vector-borne bacterial (borreliosis, brucellosis, rickettsiosis, Q-fever), protozoal diseases (malaria, leishmaniasis) as well as HIV and TB. Pathogens such as *Rickettsia* spp., *Borrelia* spp., *Leptospira* spp., *Coxiella burnetii*, and *Orientia tsutsugamushi* are of particular importance since doxycycline is a widely available and effective treatment. Clinicians should consider whether the empirical addition of doxycycline may be a suitable option. Azithromycin is a preferred alternative in areas with high burden of enteric fever due to increasing rates of quinolone resistance of *Salmonella* Typhi. Brucellosis (doxycycline dual therapy), melioidosis (ceftazidime or imipenem) and visceral leishmaniasis (liposomal amphotericin B) remain important and difficult-to-treat infections with longer combination therapy required. Our review documents scarcity of data on NTDs and this calls for additional research and improved access to diagnostics adapted to rural tropical areas, such as RDTs for enteric fever, brucellosis, leptospirosis, and rickettsiosis.

## Supporting information

S1 Prisma Checklist PRISMA 2020 Checklist. (DOCX)

S1 Table List of articles included in the final list and NIH quality assessment. (XLSX)
